# Unsatisfactory testicular position after inguinal orchidopexy: Is there a role for upfront laparoscopy?

**DOI:** 10.1080/2090598X.2019.1686895

**Published:** 2019-11-24

**Authors:** Ahmed Abdelhaseeb Youssef, Mahmoud Marei Marei, Mohamed Hamed Abouelfadl, Wesam Mohamed Mahmoud, Atef Salaheldin Abdulaziz Elbarawy, Tamer Yassin Mohamed Yassin

**Affiliations:** a Department of Pediatric Surgery, El-Demerdash Hospital, Faculty of Medicine, Ain Shams University, Cairo, Egypt; b Department of Pediatric Surgery, King Abdulaziz Medical City, Al-Hasa, Kingdom of Saudi Arabia; c Department of Surgery, Pediatric and Neonatal Surgery Unit, Cairo University Specialized Pediatric Hospital (CUSPH), Faculty of Medicine, Cairo University, Cairo, Egypt; d The Royal Manchester Children’s Hospital and Manchester Royal Infirmary, Manchester University NHS Foundation Trust, Manchester, UK; e Department of General Surgery, Faculty of Medicine, Beni-Suef University, Beni-Suef, Egypt

**Keywords:** Cryptorchidism, recurrent undescended testis, ascending testis, laparoscopic orchidopexy, redo orchidopexy

## Abstract

**Objectives**: To examine the role of laparoscopy in managing unsatisfactory testicular position after an open inguinal orchidopexy. We hypothesised that testes that were originally peeping, where short vessels represented a difficulty and testes that only reached a high scrotal position under tension, especially after an initial surgery performed with the appropriate expertise, are candidates for initial laparoscopic dissection.

**Patients and methods**: Nineteen boys with an initial open inguinal orchidopexy, with a mean age of 31 months, were considered. Twelve were then treated by a laparoscopic-assisted orchidopexy technique. Standard laparoscopy was established and utilised to mobilise the spermatic cord from above, then completed by an open inguinal mobilisation.

**Results**: The mean age at surgery was 26 months. The laparoscopic redo surgery took place at a mean interval of 11.9 months after the initial operation. The mean operative time was 72 min. A good position and size of the testis were achieved in all cases, evidenced by ultrasonography at 6 months postoperatively and clinically thereafter.

**Conclusion**: An upfront combined laparoscopic and inguinal approach to redo orchidopexy for recurrent palpable undescended testes is suitable in selected patients. This study identifies the selection criteria and outlines the operative considerations. This laparoscopic-assisted approach is a safe and feasible way to correct unsatisfactory position of the testis, with diminished risk of injury to the vas and vessels, while gaining the maximum possible length by high retroperitoneal dissection.

**Abbreviation:** UDT: undescended testis/testes

## Introduction

Cryptorchidism is the commonest urogenital congenital anomaly in males, with an incidence of 3–4% in full-term infants, reaching 30% in preterm boys and are more commonly found on the right side [1]. An undescended testis (UDT) can be clinically impalpable (20%) or palpable (80%) [2]. Within the context of age at presentation, the timing of surgery, and the situation of the contralateral testis, inguinal exploration is the mainstay of treatment for most infants with palpable UDT, reserving laparoscopy for impalpable cases and for palpable test is that cannot be brought to the scrotum without tension [3,4]. Laparoscopy for impalpable UDT was first introduced by Cortesi et al. [5] in 1976 for localisation of the testis before inguinal exploration [6,7]. Since then, laparoscopy has gained momentum and its role has expanded over the recent years [6]. Laparoscopy is currently the operation of choice for impalpable UDT.

Recurrent UDT after an inguinal orchidopexy reportedly ranges from 0.2% to 13% [8–10]. Recurrences may be classified into the failure of the testis to reach the optimal site from the outset or testicular ascent after settling at the bottom of the scrotum for some time. The exact causes of recurrence are variable and interlacing. However, failure to achieve sufficient dissection, failure of high ligation of the patent processus vaginalis, and cord structures’ entrapment within fibrotic scar tissue formation are the most commonly cited [11]. Although rare, Müllerian duct remnant structures can be the cause of the inability to achieve a tension-free orchidopexy, hence recurrence [12]. Ultrasonography is less capable than laparoscopy of detecting this particular problem and is deemed unreliable in up to 40% of cases, with evident laparoscopic superiority [13]. Moreover, shortness of the spermatic vessels is the main limiting factor, especially in peeping testes [14].

Redo orchidopexy is a challenging operation due to entrapment of the testis and spermatic cord in dense adhesions, which may jeopardise the vas deferens and testicular vessels throughout an extended inguinal dissection to allow the testis to reach the scrotum [8]. Different approaches to treat a recurrent (unsatisfactorily positioned) palpable UDT have been described, namely the inguinal approach, the scrotal approach, the totally-laparoscopic approach, and the combined approach [15–17]. However, no consensus is available regarding the selection criteria or which patients would particularly benefit from planned laparoscopy.

## Patients and methods

We reviewed the patients that were operated upon with this approach  between April 2015 and January 2018 from two tertiary paediatric surgical referral centres. Nineteen patients, with a mean age of 31 months (range 8 months – 9 years), were referred with recurrent palpable UDT after undergoing an initial open inguinal orchidopexy for palpable UDT earlier in their lives. Six patients were excluded, being candidates for a redo open inguinal surgery. One patient was excluded due to a diagnosis of atrophic testis both clinically and ultrasonographically.

We herein hypothesise that patients who underwent an open orchidopexy (especially when performed by an experienced paediatric surgeon/urologist), with one or more of the following criteria: (a) in which difficulty with short vessels was reported, (b) whose testes only reached a high scrotal position under tension after the initial surgery, and/or (c) whose testes were initially peeping, are candidates for an upfront laparoscopic dissection. On the other hand, patients who underwent an open orchidopexy done by a trainee (who may have omitted an important step during surgery), an exclusively adult-trained surgeon/urologist (who is not very familiar with the procedure), as well as those whose testis reached the bottom of the scrotum immediately postoperatively but developed gradual re-ascent with growth, should initially undergo open redo inguinal orchidopexy.

Twelve patients (with a mean age of 26 months at the time of redo surgery), with high palpable testes at the site of the previous inguinal scar or immediately below it, were treated with the laparoscopic-assisted orchidopexy technique described in this report, after reviewing their clinical and operative data in the light of our selection criteria. The operations were always performed by an experienced paediatric surgeon.

### Operative technique

The patients were placed supine under general anaesthesia. Examination under anaesthesia to detect the exact position of the testis preceded laparoscopy. Immediately before starting the procedure, the urinary bladder was emptied via a Nelaton catheter. The main surgeon stood at the contralateral side of the UDT, with the assistant on the same side (cephalad to the surgeon) and the scrub nurse at the ipsilateral side (same as the UDT). The monitor was placed at the lower end (foot-side) of the table.

**Figure 1.**
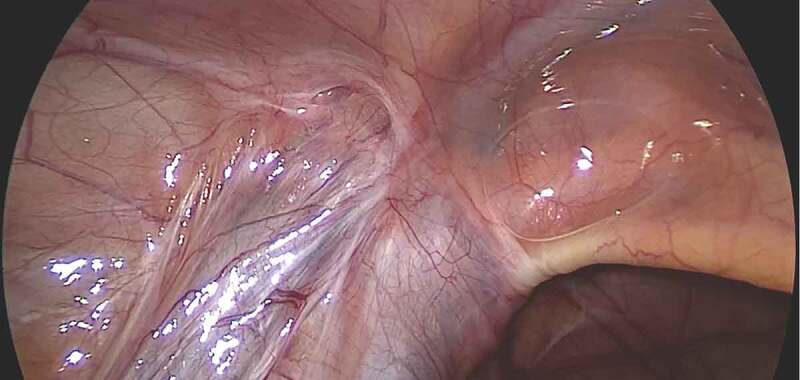
Laparoscopic view of the left deep (internal) inguinal ring showing the vas and vessels entrapped in dense adhesions.

**Figure 2.**
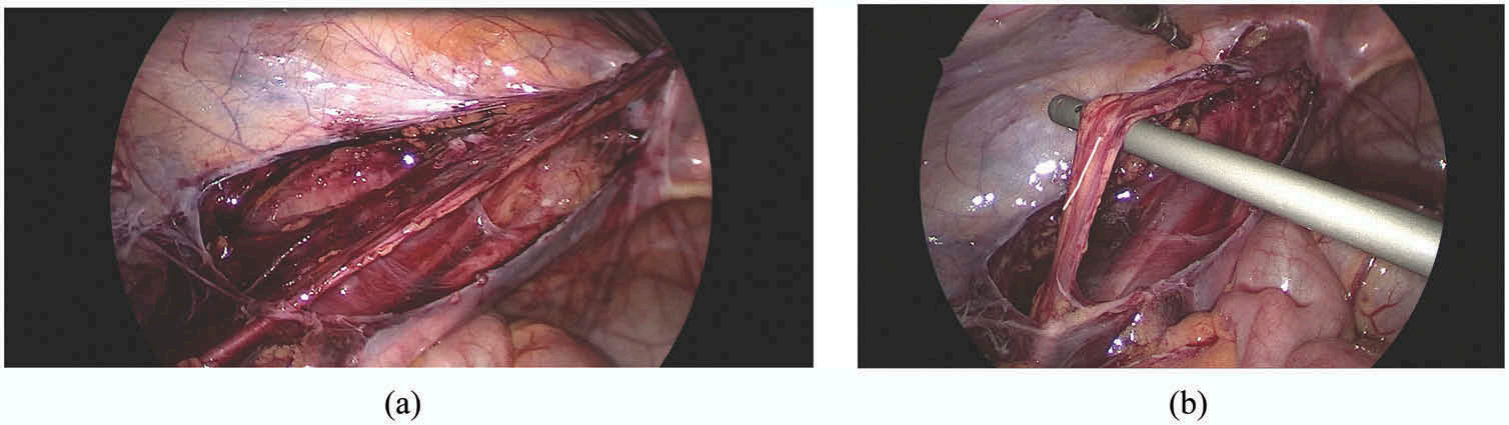
Laparoscopic operative views: (a) Dissection medial and lateral to the testicular vessels. (b) Testicular vessels gained length after dissection.

Laparoscopy was initiated through an open Hasson technique using a supra-umbilically placed 5-mm cannula. Thereafter, the peritoneal cavity was insufflated with CO_2_ (pressure: 8–12mmHg). We used a 5-mm 30 °scope and two 5-mm working ports. With the patient in the Trendelenburg position and the ipsilateral side up, the lower abdomen and pelvis were inspected to identify the vas deferens and testicular vessels entering the deep (internal) inguinal ring and to note the adhesions at the deep inguinal ring (Figure 1). Two 5-mm instruments were placed in the midclavicular line on each side, slightly infra-umbilically. The peritoneal fold containing the testicular vessels was dissected free, starting from the deep inguinal ring upwards, reaching as high as possible (Figure 2(a,b)). Extreme care was exercised during dissection of scar tissue (often in the form of a fibrotic ring) present at and around the deep inguinal ring.

**Figure 3.**
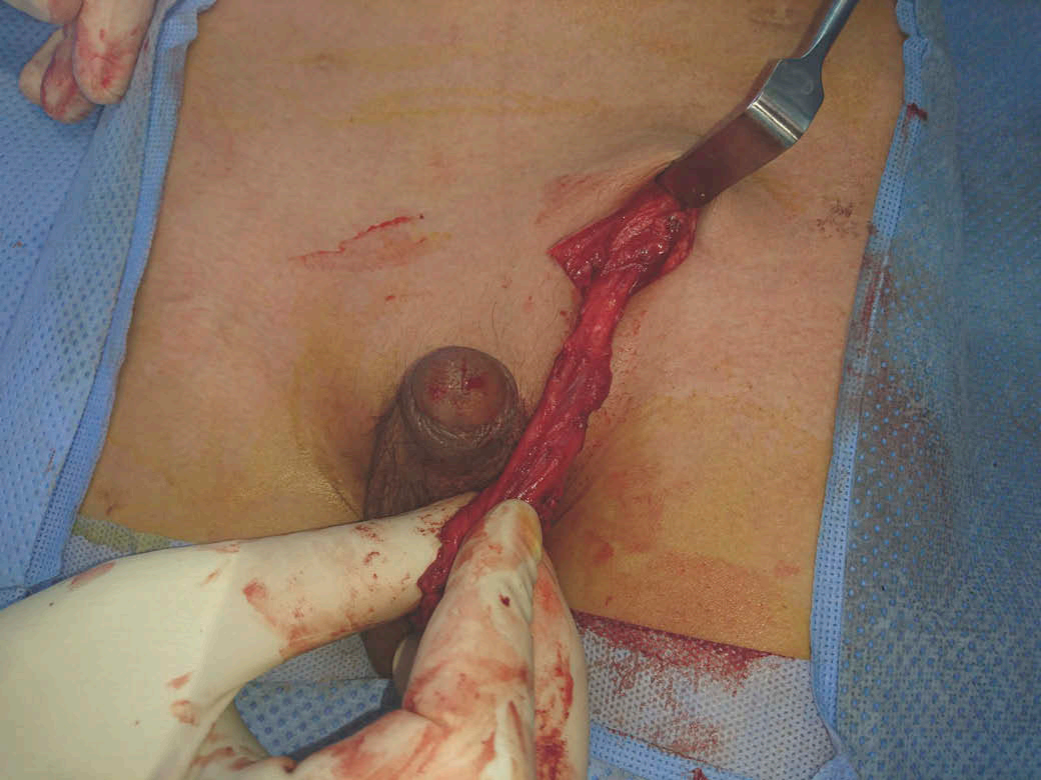
Testis reaching the scrotum easily from outside.

**Figure 4.**
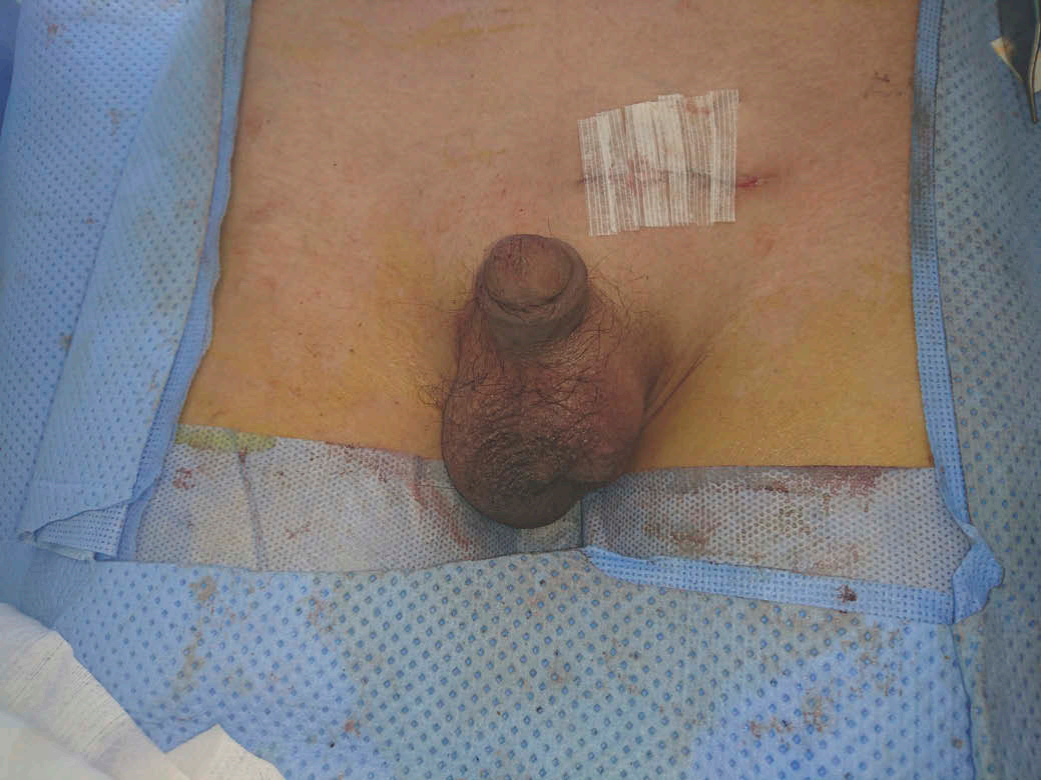
Testis fixed in the scrotum and wound closed.

The previous transverse lower inguinal crease incision was then used to gain access to the inguinal area. The incision was deepened to the external oblique aponeurosis with care, as the testis may be found in the superficial inguinal pouch. If so, the testis was freed from the external oblique aponeurosis. When the testis was proximal to the superficial (external) inguinal ring, it appeared after opening the aponeurosis. The cord was identified and then dissected free from the floor of the canal. The dissection was carried out to the deep inguinal ring; upon reaching this level, a characteristic give was noted, i.e. elongation or release of the vessels as a result of the initial laparoscopic dissection (Figure 3). In all cases, the gained length allowed the testis to reach the scrotum easily (Figure 4). Once an adequate length has been achieved, fixation of the testis to the scrotum was accomplished within a sub-dartos pouch, additionally using absorbable sutures at three points. The scrotum was then closed. The port sites and the inguinal incision were standardly closed in layers.

During all procedures, the morphology of the testis was documented, as to whether it had a normal structure to justify an orchidopexy rather than an orchidectomy.

## Results

Twelve patients with recurrent palpable UDT had an upfront combined laparoscopic and inguinal approach for redo orchidopexy. The mean age of patients at the time of surgery was 26 months (range 14 months – 9 years). The redo surgery (re-operation) took place at a mean (range) interval of 11.9 (7–24) months after the initial operation. In this series, we had more right-sided (8/12, 66.6%) cases than left-sided (4/12, 33.3%) and none were bilateral. The mean operative time was 72 min, with no intraoperative complications encountered.

Division of the spermatic vessels was not required to achieve a dependent testicular location in any of the patients included. One patient with a recurrent UDT had a remnant Müllerian structure due to persistent Müllerian duct syndrome noted upon laparoscopy, anchoring the testis, and was managed by the same technique after laparoscopic longitudinal incision (division) of the Müllerian structure.

The patients were followed-up regularly, at 1 week postoperatively then every 3–6 months until 12 months postoperatively. At their 6-month visit, a scrotal ultrasonography was performed, which confirmed a good position and size of the testis, in comparison to the contralateral one, as a reference for normality. In all 12 cases, the testes retained their scrotal position after 12 months of follow-up, and had a good size when compared to the contralateral normally descended one, by clinical examination. We considered a postoperative testicular volume of >50% on the operated side to be the least acceptable as a good outcome, which is in keeping with the published literature [18–20]. Ultrasonographically, we found the relative/differential testicular volume (operated UDT volume divided by the contralateral normal testis volume) to have a mean ratio (±SD) of 0.78 (±0.12) and a median ratio (range) of 0.8 (0.6–0.9) [18]. There was no decline in testicular size over the first postoperative year.

## Discussion

The recurrence rate after orchidopexy varies greatly in different reports (0.2–13%) [8,10]. The main cause of recurrence remains obscure [11]. Different approaches to treat a recurrent palpable UDT have been described including: the inguinal approach, the scrotal approach, the totally-laparoscopic approach, and the combined approach [15–17].

In 2011, Dudley et al [15]. published their results of the scrotal approach for recurrent palpable UDT, interestingly showing that in 11% of cases the testis could not be reached or delivered safely through a single incision and a synchronous inguinal incision was necessary. Similarly, Marret et al. [21] found that seven out of 26 cases (27%) needed a complementary inguinal incision, in addition to the scrotal approach. Consequently, the scrotal approach seems mostly suitable for high scrotal testes or testes in the superficial inguinal pouch.

Redman [11] used a unique variant of the inguinal approach, through the cremaster fascia, and had a 1.8% incidence of obvious vas injury. Besides the incision limitations, the inguinal approach might not consistently and safely offer an adequate cord length, which would ultimately lead to failure [11,17]. Traditionally, it was reported that only 37% of cases could be approached successfully by an exclusively inguinal dissection, whilst a retroperitoneal mobilisation proved necessary in up to 58% of recurrent cases [22]. The combined preperitoneal and inguinal approach described by Sfoungaris and Mouravas [17]in 2016, proved to be a safe and successful option, notwithstanding a generous incision is needed.

Similar to the situation with recurrent inguinal hernia, laparoscopy has gained a foothold, as it offers an opportunity to operate from the less fibrotic and scarred side of tissues, which is generally a common notion in re-operative surgery, in addition to a higher mobilisation and dissection of vessels. Laparoscopic magnification allows extensive and thorough dissection at the level of the deep inguinal ring, whilst preserving the vas and vessels. However, we believe that the totally-laparoscopic approach is technically demanding, and no reports comprehensively address the technicalities of testicular adhesiolysis and funiculolysis (*dissection and elongation of the spermatic cord*) [15]. Leung et al [23]. considered laparoscopy as an adjunctive step for redo inguinal orchidopexy. However, their report included only three relevant cases of redo inguinal orchidopexy, as most of their patients were primary impalpable UDT cases. In the authors’ view, this is a reasonable approach, and we would extend this even further to considering laparoscopy during the primary inguinal surgery if sufficient length is not achieved. Tong et al. [24] followed Leung’s idea but included more palpable recurrent testes (32 due to failed orchidopexies and three following testicular de-torsion of UDT), and reported a 92% success rate. The main difference between Tong’s approach and ours is that Tong reserved laparoscopy for patients whose testes failed to reach the scrotum despite complete inguinal dissection, whilst we employed an upfront laparoscopy for cases meeting our selection criteria.

Starting with inguinal surgery to mobilise the testis and considering laparoscopy only after inguinal exploration if sufficient length was not achieved, would still be our approach when no operative difficulty is anticipated beyond the specific circumstances and special situations outlined in the present report. However, this may compromise visualisation due to a pressure drop with the insufflated gas escaping through the inguinal incision, if the patent processus vaginalis was inadvertently opened, a common occurrence in redo surgeries, especially if significant inguinal dissection is needed due to fibrosis. It is also possible to deliver the testis back to the peritoneal cavity in order to redirect the spermatic vessels into a shorter route. However, we only opted to use this sparingly.

The approach described in the present study allowed redo orchidopexy to be completed easily and successfully by ensuring maximal length via intra-abdominal dissection and avoiding intraoperative injury of the cord structures by their early identification and protection. It allowed us to avoid division of the spermatic vessels, to achieve a dependent testicular location in all of the patients included, and elucidated any underlying problem that may have caused the recurrence such as a rare Müllerian remnant [12,13].

Despite the limitations of being a small cohort and the absence of a control group, our present study represents the first attempt to identify selection criteria for the use of laparoscopy after failed open orchidopexy. Methodologically, an ideal study would be a comparative one with one arm comprising our proposed approach and another arm with only inguinal surgery or inguinal surgery converted to laparoscopy if needed. This would attract a set of pertinent issues with ethical approval, inclusion criteria and possible randomisation. It could be a future direction for a subsequent study.

## Conclusion

The laparoscopic-assisted orchidopexy technique described in the present study allowed us to treat 12 patients with recurrent palpable UDT safely and without any complications. This laparoscopic-assisted approach is a safe, effective, and feasible way to correct an unsatisfactory position of the testis, potentially reducing the risk of injury to the vas and vessels, while gaining the maximum possible length by high retroperitoneal dissection. The present study identifies the selection criteria and outlines the operative considerations for this approach.

## Data Availability

The data and material for this study are available and stored confidentially.
